# Acetic Acid Bacteria in Sour Beer Production: Friend or Foe?

**DOI:** 10.3389/fmicb.2022.957167

**Published:** 2022-08-04

**Authors:** Arne Bouchez, Luc De Vuyst

**Affiliations:** Research Group of Industrial Microbiology and Food Biotechnology, Faculty of Sciences and Bioengineering Sciences, Vrije Universiteit Brussel, Brussels, Belgium

**Keywords:** sour beer, beer classification, lambic beer, *Acetobacter*, *Brettanomyces*, acetic acid bacteria

## Abstract

Beer is the result of a multistep brewing process, including a fermentation step using in general one specific yeast strain. Bacterial presence during beer production (or presence in the beer itself) is considered as bad, since bacteria cause spoilage, produce off-flavors, and/or turbidity. Although most problems in the past related to lack of hygiene and/or cleaning, bacteria do still cause problems nowadays. Despite this negative imago, certain bacteria play an irreplaceable role during fermentation and/or maturation of more unique, funky, and especially refreshing sour beers. The term *sour beers* or *sours* is not restricted to one definition but covers a wide variety of beers produced *via* different techniques. This review proposes an uncluttered sour beer classification scheme, which includes all sour beer production techniques and pays special attention to the functional role of acetic acid bacteria. Whereas their oxidation of ethanol and lactate into acetic acid and acetoin usually spoils beer, including sour beers, organoleptically, a controlled growth leads to a desirable acidic flavor in sour beers, such as lambic-style, lambic-based, and red-brown acidic ales.

## Introduction

Food fermentations have been done by humans for thousands of years as means of preservation of raw materials from agricultural and husbandry origin (Hutkins, 2019). Other desirable attributes of fermented food products, such as unique flavors, textures, appearances, or other functionalities were recognized rapidly as well (Leroy and De Vuyst, 2004). With the development of other preservation techniques, a lot of fermentation processes have been replaced, and the main goal of the production of fermented foods has shifted from preservation to flavor production and health promotion (Marco et al., 2021). Also, food fermentations are associated with cultural connotations, gastronomic qualities, artisan characteristics, and natural appeal. In several food fermentation processes, not only yeasts and lactic acid bacteria (LAB) but also acetic acid bacteria (AAB) are involved, such as in the production of cocoa, kombucha, lambic beer, vinegar, and water kefir (Gullo et al., 2016; Pothakos et al., 2016; Cousin et al., 2017; De Roos and De Vuyst, 2018; Gomes et al., 2018; Villareal-Soto et al., 2018; De Vuyst and Leroy, 2020; Bongaerts et al., 2021; Lynch et al., 2021).

Fermented foods and drinks play a major role in the human diet and human nutrition worldwide (Marco et al., 2017, 2021). Beer, the end-product of the brewing process, including a fermentation and maturation step, is the most consumed fermented beverage by humans worldwide (Neves et al., 2011; Colen and Swinnen, 2015). Whereas, originally, beer was fermented in a spontaneous way, due to lack of knowledge and starter cultures, all beers were at least slightly acidic (Hornsey, 2003). This acidity contributed to a safe water supply for beer drinkers, as hop and spice antimicrobial compounds do for non-sour beers. Nowadays, for almost all beers produced the fermentation relies on specific yeast strains and the presence of bacteria is completely undesirable due to their spoilage potential (Briggs et al., 2004; Vriesekoop et al., 2012). However, although their spoilage capacity, some specific beers do require LAB and/or AAB to introduce characteristic beer flavors (Van Oevelen et al., 1977; De Keersmaecker, 1996; Tonsmeire, 2014; De Roos and De Vuyst, 2019; Bongaerts et al., 2021; Kubizniaková et al., 2021). Sour beers, with their typical refreshing and (slightly) acidic flavor because of high organic acid concentrations are an example of such beers, during the production process of some LAB or even both LAB and AAB are part of the core microbiota and hence contribute to their flavor formation (Van Oevelen et al., 1976; Snauwaert et al., 2016; De Roos and De Vuyst, 2019; Bongaerts et al., 2021). Today, the production of sour beers or sours knows an increasing trend.

## Beer Spoilage by AAB

AAB are traditionally well known for their beer-spoiling capacity. Beer spoilage has been a problem since multiple decades (Sakamoto and Konings, 2003). The combination of multiple inhibitory factors or hurdles, such as the presence of ethanol (up to 10%, v/v), hop antimicrobial compounds, a low pH, relatively high carbon dioxide concentrations, low oxygen concentrations, and the lack of nutritive compounds makes beer hard to spoil (Kourtis and Arvanitoyannis, 2001; Sakamoto and Konings, 2003; Briggs et al., 2004; Menz et al., 2009; Dysvik et al., 2020c). Despite this harsh environment, some Gram-positive bacteria, Gram-negative bacteria, and so-called wild yeasts are capable of spoiling beer (Van Vuuren et al., 1979; Vriesekoop et al., 2012; Schneiderbanger et al., 2020; Suiker and Wösten, 2021). The Gram-negative bacteria capable of beer spoilage include enterobacteria (such as *Citrobacter*, *Klebsiella*, *Rahnella*, and *Obesumbacterium*), *Zymomonas* spp., *Pectinatus* spp., *Megasphaera* spp., *Selenomonas* spp., *Zymophilus* spp., and AAB, whereof *Acetobacter* and *Gluconobacter* have been mainly reported (Sakamoto and Konings, 2003; Van Vuuren and Priest, 2003). Even though AAB species are strict aerobic, some strains have been detected and identified from beer and have been reported as micro-aerotolerant (Harper, 1980; Briggs et al., 2004; Jeon et al., 2014). Today, aerobic AAB species do not form a big problem of beer spoilage anymore thanks to the development of improved brewing technology and beer storage, capable of lowering oxygen levels drastically (Sakamoto and Konings, 2003).

As AAB are in particular acid- and ethanol-tolerant and not inhibited by hop compounds, they may grow in beer. Beer spoilage by AAB species is characterized by a sour taste and vinegary aroma, caused by ethanol oxidation into acetic acid (Ingledew, 1979; Magnus et al., 1986). Besides off-flavor formation, AAB species, such as *Acetobacter aceti, Acetobacter liquefaciens*, *Acetobacter pasteurianus, Acetobacter hansenii* and *Gluconobacter oxydans*, can cause haziness and ropiness in the beer or form pellicles on the beer surface (Van Vuuren, 1999; Van Vuuren and Priest, 2003; Briggs et al., 2004; Hill, 2015; Paradh, 2015).

Since most spoilage incidents with AAB are related to oxygen, the key to prevent spoilage by AAB is to limit oxygen ingress as much as possible and apply good hygiene regimes. Additional care should be applied during bottling in the brewery and cleaning of beer lines, taps, and dispense systems in pubs (Briggs et al., 2004; Vriesekoop et al., 2012). Alternatively, when AAB belong to the desired fermentation microbiota, key will be to have their growth under control.

## General Beer Classification

Beers are generally classified within four different beer production types, being bottom-fermented beers, top-fermented beers, spontaneously fermented beers, and mixed-fermented beers (Briggs et al., 2004; De Roos and De Vuyst, 2019; Bongaerts et al., 2021). This classification is made according to the fermentation step and which microorganisms are involved, in particular related to their origin. The use of specific strain(s) of the yeast species *Saccharomyces bayanus* or *Saccharomyces pastorianus* for bottom fermentation and *Saccharomyces cerevisiae* for top fermentation characterizes these yeast-based fermentation processes, which are carried out in stainless-steel vessels. In stark contrast to these very controlled fermentation processes stands a spontaneous one, for which a diverse multistage fermentation and maturation process in horizontal wooden barrels results in a unique sour beer, thanks to the successive activities of different microbial groups, in particular yeasts, LAB, and AAB (Bokulich et al., 2012; Spitaels et al., 2014a, 2015b; De Roos et al., 2018a,b, 2020; De Roos and De Vuyst, 2019; Bongaerts et al., 2021). Mixed fermentation is a combination of top and spontaneous fermentation techniques, for which process an in-house starter culture, a yeast slurry also containing LAB from previous fermentations, is added, and maturation in vertical wooden barrels (potentially involving AAB) or stainless-steel vessels (Tonsmeire, 2014; Snauwaert et al., 2016; Spitaels et al., 2017; De Roos and De Vuyst, 2019).

## Extended Classification System

The traditional classification into four different beer production types does not cover all beers on the market nowadays, especially more experimental beers, craft beers, or beers produced by microbreweries, which are known to experiment more in the search for new flavor profiles. The craft beer industry, growing since the 1970s, is characterized not only by reusing traditional techniques and brewing with traditional ingredients but also by their diverging application regarding ingredients used, yeasts applied, alcohol content, aging, isotonic claims, and/or packaging (Donadini and Porretta, 2017; Li et al., 2017; Baiano, 2020; Lattici et al., 2020).

**Figure 1:**
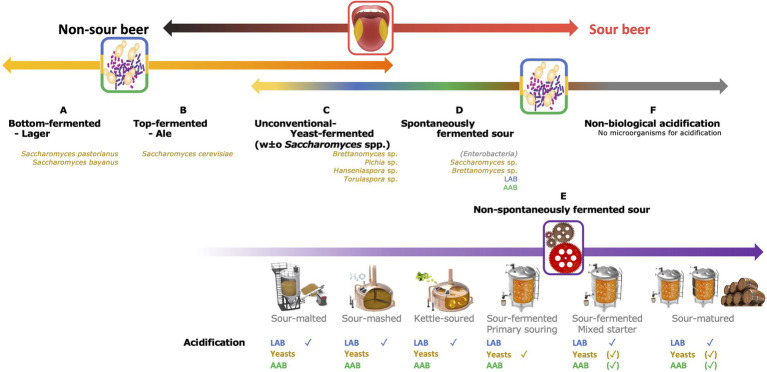
Schematic representation of an extended beer classification. On top, a first subdivision is made based on taste [from left to right non-sour (black) to sour (red)]; in the middle, distinction is made based on the fermentation technology applied and the microorganisms involved [yeasts (yellow-orange); lactic acid bacteria (LAB, blue); acetic acid bacteria (AAB, green); and no microorganisms (gray)]. Category E is further subdivided according to the acidification technique applied and the microorganisms responsible for the acidification are indicated.

The traditional four-type classification system hence only includes three added *Saccharomyces* species, namely *S. cerevisiae* for top fermentation and *S. bayanus* or S*. pastorianus* for bottom fermentation. Therefore, beers produced using other, non-*Saccharomyces* yeast species, such as *Brettanomyces* spp., *Torulaspora* spp., *Pichia* spp., etc., cannot be classified unambiguously. Consequently, an extended classification system is suggested in this review, taking into account the fermentation type (distinguishing five production processes), the microorganisms involved (not only yeasts but also LAB and AAB), and the acidification principle (Figure 1). A separate beer production type, indicated as non-conventional fermented beers, covers all beers fermented solely using yeasts, except for *S. cerevisiae*, *S. pastorianus* and *S. bayanus* (Type C in Figure 1). This beer production type likely makes the transition from yeast-based fermentation processes to sour beer production types.

Whereas non-sour beers rely on fermentation and/or maturation steps done using an axenic *Saccharomyces* yeast strain, the story differs for sour beers (Bokulich and Bamforth, 2013; Tonsmeire, 2014; Bossaert et al., 2019; Dysvik et al., 2020c).

### Spontaneously Fermented Sour Beers

One of the, if not the, most traditional sour beer production processes is based on spontaneous inoculation, thus without initiation of the fermentation of the wort by addition of a starter culture (Type D in Figure 1). It mainly concerns Belgian lambic beer and American coolship ale (ACA) productions, two of the most popular spontaneously fermented beers worldwide. Generally, the boiled and sterile wort is inoculated *via* multiple ways with a so-called wild microbiota, consisting of both wanted and unwanted yeasts and bacteria (De Keersmaecker, 1996; Spitaels et al., 2014a, 2015b; De Roos and De Vuyst, 2019; Bongaerts et al., 2021). Inoculation takes place when the environmental air gets into contact with the wort during an overnight cooling step using a metallic open vessel or coolship, when the wort gets into contact with the surfaces of the brewery equipment, and especially by contact of the fermenting wort and maturing beer with the interior surfaces of the horizontal, wooden barrels during the long-lasting fermentation and maturation steps (De Keersmaecker, 1996; De Roos and De Vuyst, 2019; Bongaerts et al., 2021). Therefore, spontaneously fermented beers are produced during the winter period to cool down the boiled wort fast enough, and so limit enterobacterial growth, in particular when the wort is not acidified manually, and in temperature-controlled cellars to achieve an optimal microbial succession (Bokulich et al., 2012; De Roos et al., 2018a; De Roos and De Vuyst, 2019; Bongaerts et al., 2021).

Specifically for bacteria, a broad range of species, including enterobacteria, LAB, and AAB have been isolated and identified from spontaneously fermenting lambic beer and ACA worts (Bokulich et al., 2012; De Roos and De Vuyst, 2019; Bongaerts et al., 2021; De Roos et al., unpublished results). Bacteria are typically present from the start of the lambic beer wort fermentation till the end of the extended barrel maturation process, which can last up to 3 years (Van Oevelen et al., 1977; Verachtert and Iserentant, 1995; De Roos et al., 2018b; Bongaerts et al., 2021). They are also present in lambic-based beers, such as gueuze, a bottle-refermented blend of young and old lambic beers (Spitaels et al., 2015a; De Roos et al., 2018a,b, 2020; Bongaerts et al., 2021; Piraine et al., 2021). More specifically, AAB are encountered during nearly the whole fermentation and maturation process of lambic beers but are mainly active during the first weeks, called the wild or enterobacterial and thus initial fermentation phase, during the acidification phase (two up to 12 months), and during maturation (up to 3 years; Bokulich et al., 2012; Spitaels et al., 2014a; De Roos and De Vuyst, 2019; Bongaerts et al., 2021). Next to gueuze, different types of lambic-based sour beers are produced in a traditional way, in particular fruit beers, such as *Oude Kriek* [additional fermentation in barrels of young (around 1 year old) lambic beer blended with fresh sour cherries], *Framboise* (young lambic beer blended with raspberries), and *Pecheresse* (young lambic beer blended with peaches), as well as Faro (young lambic beer blended with rock sugar; De Keersmaecker, 1996; Briggs et al., 2004; Tonsmeire, 2014; Verachtert and Derdelinckx, 2014; Pothakos et al., 2016; Dysvik et al., 2020c).

Culture-dependently, AAB species belonging to only two genera (*Acetobacter* and *Gluconobacter*) have been isolated and identified from spontaneously fermented sour beers (Bokulich et al., 2012; Spitaels et al., 2014a,b,c, 2015a,b; Snauwaert et al., 2016; De Roos et al., 2018a,b; Table 1). Culture-independent methods, such as metagenetics (targeting a part of or the whole 16S rRNA marker gene) and shotgun metagenomics, have led to the identification of not only *Acetobacter* spp. and *Gluconobacter* spp. but also *Gluconacetobacter* spp. and *Komagataeibacter* spp. (Bokulich et al., 2012; Snauwaert et al., 2016; De Roos et al., 2018a, 2020; Piraine et al., 2021; Tyakht et al., 2021; De Roos et al., unpublished results; Table 2).

**Table 1:** Overview of acetic acid bacterial species identified culture-dependently from sour beers.

**Taxon**	**Production phase**				**Isolation medium**	**Reference**
	Initial fermentation phase	Alcoholic fermentation phase	Acidification phase	Maturation phase		
**Belgian lambic beer**						
*Acetobacter cerevisiae*	Inside cask ✓ ✓				AAM mDMS mDMS	Spitaels et al., 2014a De Roos et al., 2018b De Roos et al., unpublished results
*Acetobacter orientalis*	✓ ✓ ✓	✓	✓		mMRS mDMS AAM	De Roos et al., 2018a De Roos et al., 2018b Spitaels et al., 2015a
*Acetobacter lambici*	✓	✓	✓ ✓	✓ ✓ ✓ ✓	mDMS mMRS AAM AAM AAM mDMS	De Roos et al., 2018b De Roos et al., 2018a Spitaels et al., 2015a Spitaels et al., 2014c Spitaels et al., 2015b De Roos et al., unpublished results
*Acetobacter pasteurianus*			✓	✓	mDMS	De Roos et al., 2018b
*Acetobacter aceti*	✓				mDMS	De Roos et al., 2018b
*Acetobacter lovaniensis*	✓				mDMS	De Roos et al., 2018b
*Acetobacter indonesiensis*	✓				mDMS	De Roos et al., 2018b
*Acetobacter fabarum*	✓ ✓	✓	✓	✓	AAM mDMS	Spitaels et al., 2015a De Roos et al., unpublished results
*Gluconobacter cerevisiae*	✓ ✓	✓	✓		mDMS AAM	De Roos et al., 2018b Spitaels et al., 2014b, 2015a
*Gluconobacter wancherniae*	✓				mDMS	De Roos et al., 2018b
*Gluconobacter cerinus*	✓	✓			AAM	Spitaels et al., 2015a
**American coolship ales**						
*A. fabarum*		✓	✓	✓	WLD	Bokulich et al., 2012
*Acetobacter lovanesiensis*	✓	✓	✓	✓	WLD	Bokulich et al., 2012
**Belgian red-brown acidic ales**						
*A. pasteurianus*				✓	mDMS	Snauwaert et al., 2016

*AAM, acetic acid medium; mDMS, modified deoxycholate-mannitol-sorbitol medium; mMRS, modified de Man-Rogosa-Sharpe medium; and WLD, Wallerstein differential medium*.

**Table 2:** Overview of acetic acid bacterial species identified culture-independently from sour beers.

**Taxon**	**Production phase**				**Technique**	**Reference**
	Initial fermentation phase	Alcoholic fermentation phase	Acidification phase	Maturation phase		
**Belgian lambic beer**						
*Acetobacter* spp.	✓ ✓	✓	✓ ✓ ✓	✓ ✓ ✓	Metagenetics (V4 region of 16S rRNA gene) Shotgun metagenomics Shotgun metagenomics	De Roos et al., 2018a De Roos et al., 2020 De Roos et al., unpublished results
*Acetobacter cerevisiae*	✓				Shotgun metagenomics	De Roos et al., unpublished results
*Acetobacter fabarum*	✓				Shotgun metagenomics	De Roos et al., unpublished results
*Acetobacter indonesiensis*	✓				Shotgun metagenomics	De Roos et al., unpublished results
*Acetobacter lambici*				✓	Shotgun metagenomics	De Roos et al., unpublished results
*Acetobacter malorum*	✓				Shotgun metagenomics	De Roos et al., unpublished results
*Acetobacter pasteurianus*			✓		Shotgun metagenomics	De Roos et al., 2020
*Acetobacter pomorum*			✓		Shotgun metagenomics	De Roos et al., 2020
*Gluconobacter* spp.	✓		✓	✓	Shotgun metagenomics Shotgun metagenomics	De Roos et al., 2020 De Roos et al., unpublished results
	✓				Shotgun metagenomics	De Roos et al., 2018a
*Gluconobacter japonicus*	✓				Shotgun metagenomics	De Roos et al., unpublished results
*Gluconacetobacter* spp.			✓	✓	Shotgun metagenomics	De Roos et al., 2020
*Gluconacetobacter liquefaciens*	✓				Shotgun metagenomics	De Roos et al., unpublished results
*Komagataeibacter* spp.			✓	✓	Shotgun metagenomics	De Roos et al., 2020
**American Coolship Ales (ACAs)**						
*Acetobacteraceae*		✓		✓	16S-Terminal Restriction Fragment Length Polymorphism (TRFLP)	Bokulich et al., 2012
**Belgian red-brown acidic ales**						
*Acetobacteraceae*	Bottled red-brown acidic ale				Metagenetics (V4 region of 16S rRNA gene)	Tyakht et al., 2021
*A. pasteurianus*				✓	Metagenetics (V1-V3 region of 16S rRNA gene)	Snauwaert et al., 2016
**Spontaneously fermented beer**						
*Acetobacter* spp.	Finished beer, beer slurry				Metagenetics (V3-V4 region of 16S rRNA gene)	Piraine et al., 2021
*Gluconobacter oxydans*	Finished beer				Metagenetics (V3-V4 region of 16S rRNA gene)	Piraine et al., 2021

### Non-spontaneously Fermented Sour Beers

Multiple techniques of sour beer production fall in this category, differing according to the acidification method and especially where and when the acidification takes place (Bossaert et al., 2019; Dysvik et al., 2020a,b,c; Type E in Figure 1).

#### Sour-Malted and Sour-Mashed Beers

Malt, barley grains processed during the malting process, is used for almost all beers produced worldwide (Briggs et al., 2004). In the case of sour malting and sour mashing, acidification takes place during malting or mashing, respectively (Bossaert et al., 2019; Dysvik et al., 2019, 2020b,c). The acidification is achieved by the growth and metabolic activity of LAB, such as *Lactiplantibacillus plantarum* or *Pediococcus pentosaceus* (Laitila et al., 2006; Vriesekoop et al., 2012; Peyer et al., 2017). Carbohydrates present on the surfaces of the malted grains or in the mash are converted into lactic acid and in the case of sour malting, this lactic acid is retained on the grain surfaces (Lowe and Arendt, 2004; Vriesekoop et al., 2012). Lactic acid typically lowers the pH value around 0.15 to 0.25 units and therefore only 3–10% of the total grist will be acidified malt (Lowe et al., 2004; Lowe and Arendt, 2004). The resulting wort using acidified malts has a pH value around 5.2, in contrast with a pH of around 5.5 for non-acidified malt-based wort (Back, 2005; Vriesekoop et al., 2012). Whereas the influence of LAB starter cultures seems marginally, it results in an improved malting process, by suppressing rootlet growth of the germinating grain kernels, which has been shown to outperform chemical rootlet inhibitors (Vriesekoop et al., 2012). LAB starter culture application during the malting process results in an increased malt yield, an improved filterability, and lower wort viscosity (Vriesekoop et al., 2012). Additionally, LAB show certain antifungal and antibacterial activities, lowering the growth of fungi, such as *Fusarium*, which are involved in gushing and potential mycotoxin production (Batish et al., 1997; Lowe and Arendt, 2004; Shetty and Jespersen, 2006; Rouse et al., 2008; Vriesekoop et al., 2012).

#### Kettle-Soured Beers

During kettle souring, LAB acidify the wort in the brewing kettle. Sometimes, wort is boiled (without hops) prior to LAB pitching or the LAB inoculation can happen straight after mashing, without boiling (Cantwell and Bouckaert, 2016; Bossaert et al., 2019; Dysvik et al., 2020c). Kettle souring, also called quick souring, is a modern technique with the biggest advantage that the desired acidification typically takes place after one to 3 days (Bossaert et al., 2019). Acidification can be stopped either by boiling or by the addition of (heavily) hopped wort. LAB are then inactivated due to their sensitivity to hop-related compounds, such as 𝛼-acids (humulones), 𝛽-acids (lupulones), or their isomerized forms (iso-𝛼-acids or iso-humulones and iso-𝛽-acids or iso-lupulones, respectively; Almaguer et al., 2014). Further, addition of heavily hopped wort limits the loss of flavor compounds, which can happen during boiling (Tonsmeire, 2014; Bossaert et al., 2019; Dysvik et al., 2020c). Yet, when a less complex sour beer is wanted, boiling after acidification can be desired (Tonsmeire, 2014; Admassie, 2018).

#### Sour-Fermented Beers

Sour-fermented beers comprise all techniques for which ethanol production and acidification take place at the same moment. Sour-fermented beers can be divided into two large categories, based on the fact if there are bacteria present or not. For both, wort is prepared as usual, and the fermentation takes place in stainless-steel vessels (Tonsmeire, 2014).

##### Primary Souring

Sour-fermented beers produced in the total absence of any bacteria must be produced through fermentation with yeast species capable of degradation of carbohydrates into lactic acid, ethanol, and carbon dioxide (Osburn et al., 2016, 2018). *Hanseniaspora vineae, Lachancea fermentati, Lachancea thermotolerans, Schizosaccharomyces japonicus,* and *Wickerhamomyces anomalus* species have been tested before and most strains examined are able to completely ferment the wort within 4 weeks (Domizio et al., 2016; Osburn et al., 2018; Bossaert et al., 2019). Whereas these yeast species have mainly been reported as members of mixed-starter cultures for beer wort and wine must fermentations, generally combined with *Saccharomyces* yeast species, they are able to attenuate beer wort sufficiently when used as the sole yeast starter, and they are capable of producing L-lactic acid in sufficient concentrations to produce sour beers (Banilas et al., 2016; Domizio et al., 2016; Benito, 2018; Osburn et al., 2018; Larroque et al., 2021; Vicente et al., 2022). Strain selection is of major importance, since large within-species and -strain variations occur, which greatly influences the final products (Osburn et al., 2018; Gatto et al., 2020). A high variability has been reported regarding lactic acid and ester production, flavor formation, sourness perception, and final pH (Domizio et al., 2016; Gamero et al., 2016; Osburn et al., 2018; Gatto et al., 2020; Vicente et al., 2021). Brewing primary-soured beers has the additional advantage of being produced without blocking the brewing kettle for days. It results in better sensory profiles compared to kettle-soured beers, without a long barrel aging process as is the case for spontaneously fermented sour beers (Osburn et al., 2018). The absence of acidifying bacteria in the brewing apparatus and brewery eliminates the risk of contaminating non-sour beers, especially when both sour and non-sour beers are brewed on the same site and/or using the same brewing equipment (Osburn et al., 2018; Bossaert et al., 2019). Additionally, yeast growth is very limited or completely not impacted by the hop dosing and release of iso-𝛼-acids into the wort. Consequently, higher hop dosages can be applied during primary souring brewing, since acidification does not rely on bacteria, which are generally more sensitive to iso-𝛼-acids, in particular Gram-positive LAB (Hazelwood et al., 2010; Almaguer et al., 2014; Domizio et al., 2016; Osburn et al., 2018).

##### Mixed-Culture Fermented Sour Beers

The term mixed culture is used when more than one specific microbial strain and/or species is present during fermentation. The application of a mixed-starter culture differs from spontaneously fermented beers, such as lambic beer, in that in the latter case all yeasts and bacteria originate from the environment and/or brewing tools (De Keersmaecker, 1996; De Roos et al., 2018b; Bongaerts et al., 2021). Both traditional, red/brown (Flanders red ales) and old-brown (Flanders brown ales) acidic (Flemish) ales, and modern mixed-culture sour beers (Flanders-style sour ales) exist on industrial scale nowadays (Tonsmeire, 2014; Snauwaert et al., 2016; Bossaert et al., 2019; Dysvik et al., 2020c). During red/brown acidic ale production, a yeast slurry from previous fermentation processes is added to the cooled wort to perform the fermentation. Although the yeast slurry typically undergoes an acid wash, it still contains LAB (Martens et al., 1997). Initially, AAB have not been detected in the slurry or during primary fermentation (Martens et al., 1997). However, making use of appropriate selective agar media and incubation conditions, AAB have been isolated from beers at the end of the maturation phase, in particular *A. pasteurianus* (Snauwaert et al., 2016). Notice that the final beers representing Flanders red ales are also blends of two-year barrel-matured beers and young, non-matured beers, whereby different blend ratios give different red-brown sours (such as Rodenbach Classic, Grand Cru, and Vintage). During modern mixed fermentation processes, a mixture of yeasts (*Saccharomyces* and/or non-*Saccharomyces* spp.) and bacteria (LAB and/or AAB) is added as starter culture after wort production, either all at the same time, or spread over time (Peyer et al., 2017; Ciosek et al., 2019; Dysvik et al., 2019, 2020a,b,c).

#### Sour-Matured Beers

When acidification happens during the maturation phase, either solely during maturation or as further souring during maturation, the beers can be classified as sour-matured. Sour maturation can take place in both stainless-steel vessels or wooden barrels, but maturation differs according to the container used (Tonsmeire, 2014; Snauwaert et al., 2016).

##### Sour Maturation in Wooden Barrels

Wooden barrels were historically by far the most applied beer transport and storage tool, but have later disappeared due to practical reasons and the unpredictability of the quality of the resulting beers (Twede, 2005; Tonsmeire, 2014; Bossaert et al., 2019, 2020, 2022b). Yet, wooden barrels are nowadays gaining interest again, either for production or maturation, to introduce additional flavors and hence achieve more complex flavor profiles and/or sour beers (Garcia et al., 2012; Tonsmeire, 2014; Cantwell and Bouckaert, 2016; Bossaert et al., 2019, 2020; Shayevitz et al., 2020). Although introducing wood-associated flavors seems to be the most obvious reason, wooden barrel maturation does more. Flavors from previous uses of the barrels, such as for the production of port wine, wine or spirits, or for storage, can be introduced in the maturing beer (De Rosso et al., 2009; Fernandez de Simon et al., 2014; Cantwell and Bouckaert, 2016; Shayevitz et al., 2020). Also, microbial activity during barrel maturation can lead to new flavors, including a sour taste and acidic notes (Tonsmeire, 2014; Cantwell and Bouckaert, 2016; Bossaert et al., 2019, 2022a). Wooden barrels are generally made of oak, chestnut, cherry, and/or acacia wood (Cantwell and Bouckaert, 2016; Bossaert et al., 2019, 2020, 2022c). Due to the porous nature of the wood, wooden barrels are slightly permeable for oxygen gas and thus create a microaerobic environment, which may allow the growth of AAB (Cantwell and Bouckaert, 2016; De Roos et al., 2019; Bongaerts et al., 2021). Also linked with their porosity, wooden barrels harbor microorganisms up to 1.2 cm deep and act so as inoculation source for the fermenting wort and/or maturing beer (De Roos et al., 2019; Shayevitz et al., 2020; Bongaerts et al., 2021). The typical wooden barrel-associated microbiota consists of *Brettanomyces* yeasts, LAB and AAB, which have been isolated numerously from barrel-aged beers, including barrel-aged ales (Bokulich et al., 2012; Spitaels et al., 2014a; De Roos et al., 2018b, 2019; Shayevitz et al., 2020). In combination with a microaerobic environment, ethanol can be oxidized to acetic acid easily, which in turn impacts the beer flavor significantly and causes acidification of the beer (Cantwell and Bouckaert, 2016; De Roos et al., 2019; Shayevitz et al., 2020). Despite widespread use of wooden barrels, barrel maturation is generally a long-lasting process, and it remains trial and error concerning the flavor of the final beer, since the outcome relies on many factors including barrel characteristics, such as barrel history, barrel cleaning methods applied, barrel condition, and barrel wood, intrinsic beer characteristics such as alcohol level and pH, duration of the maturation, temperature, and humidity (Garcia et al., 2012; Sterckx et al., 2012; Cantwell and Bouckaert, 2016; Bossaert et al., 2022a,c).

##### Sour Maturation in Metallic Vessels

In contrast with wooden barrels, stainless-steel vessels are not permeable for oxygen gas and do not harbor microorganisms. Consequently, acidification in metallic vessels can take place if the beer itself contains acidifying microorganisms, such as LAB, or acidifying microorganisms can be added (Tonsmeire, 2014). One of the most known beers produced through acidification in metallic vessels are old-brown acidic ales (Flanders brown ales; Tonsmeire, 2014; De Roos and De Vuyst, 2019). Old-brown acidic ales are produced as red/red-brown acidic ales but differ in the usage of metallic vessels for old-brown acidic ales and wooden barrels for red/red-brown acidic ales, and have been described as more malty and less acidic (Verachtert and Iserentant, 1995; Martens et al., 1997; Preedy, 2008; Tonsmeire, 2014; Snauwaert et al., 2016; De Roos and De Vuyst, 2019). The sour taste and acidic notes of old-brown acidic ales mainly comes from LAB activity during fermentation, prior to the metallic vessel maturation, but these beers do not acidify by acetic acid formation during maturation (Tonsmeire, 2014; Snauwaert et al., 2016). This must be ascribed to the lack of inoculation of barrel-associated AAB and the anaerobic environment inside stainless-steel vessels.

### Non-biological Acidification

Whereas all beers of Types D and E are acidified by bacteria and/or yeasts, sour beers can also be acidified without microbial interference (Tonsmeire, 2014; Dysvik et al., 2019; Tan et al., 2021; Type F in Figure 1). Non-biologically acidified beers, also called chemically acidified beers, are produced by adding food-grade organic acids, such as lactic acid, fresh fruits, fruit juices, or lemonades (Franz et al., 2009; Tonsmeire, 2014; Dysvik et al., 2019; Tan et al., 2021). The main advantage linked with this production technique is the ability to experiment extensively with juice/beer ratios or the kinds of fruits used (Tonsmeire, 2014). In general, the most used fruits are berries, such as blueberries and raspberries, and cherries, but many more have been used, such as citrus fruits, peaches, mangoes, etc. (Tonsmeire, 2014; Tan et al., 2021). Beers produced by blending fruit juices with finished (eventually pasteurized) beers should not be confused with traditional fruit lambic beers, as the latter still evolve over time by the presence of metabolically active microorganisms, whereas non-biological acidified beers do not evolve anymore.

## Functional Role of AAB During Sour Beer Production

AAB, and bacteria in general, are completely unwanted during non-sour beer production, mainly due to their acetic acid production turning the beer into vinegar (Bokulich and Bamforth, 2013). Despite their bad reputation, AAB do contribute unambiguously to desired flavor formation during the fermentation and/or maturation of certain sour beer types (Vriesekoop et al., 2012; De Roos and De Vuyst, 2019; Bongaerts et al., 2021; Figure 1). As mentioned above, the best studied example of AAB in beers is their appearance and functionality during fermentation and maturation of spontaneously fermented beers, such as lambic beer, and to a lesser extent the ACA analogue (Martens et al., 1997; Bokulich et al., 2012; Spitaels et al., 2014a, 2015b, 2017; Tonsmeire, 2014; Snauwaert et al., 2016; De Roos and De Vuyst, 2019; Bongaerts et al., 2021). Historically, the functional role of AAB in spontaneously fermented beers was considered limited and solely restricted to the oxidation of ethanol to acetic acid. Although this is their most impacting and characterizing feature, more functionalities and contributions during fermentation and maturation have been described in the last decade. Limited literature is available about AAB presence in sour beers, except for red-brown acidic ales, ACAs and especially lambic beer, and so is the following writing mainly based on findings during spontaneously fermented beers, and to a lesser extent on findings originating from research applied on red-brown acidic ales.

### Acetic Acid Production

Acetic acid bacteria occurrence in beers is mainly linked with their most characterizing feature, being the aerobic, incomplete oxidation of ethanol, carbohydrates, or sugar alcohols by dehydrogenase activities into the corresponding organic acids, sometimes referred to as oxidative fermentation (Cleenwerck et al., 2002; Ashtavinayak and Elizabeth, 2016; De Roos and De Vuyst, 2018; De Roos et al., 2018a). The two-step catalytic oxidation of ethanol comprises first oxidation of ethanol by membrane-bound pyrroloquinoline quinone (PQQ)-dependent alcohol dehydrogenase (ADH) activity to acetaldehyde and the further oxidation of the latter compound by a membrane-bound aldehyde dehydrogenase (ALDH) to acetic acid (Yakushi and Matsushita, 2010; Gomes et al., 2018). Since both enzymes, ADH and ALDH, form a multienzyme complex, acetaldehyde is not released (Gomes et al., 2018). Acetic acid influences the beer flavor in a drastic way, since it is described as harsh, and it thus contributes a sharp sourness and vinegary notes if present above the threshold concentration of around 200 mg/l (Van Oevelen et al., 1976; De Roos et al., 2019). Although acetic acid possibly causes problems in non-sour beers or when it is present in excessively high concentrations in sour beers, it is crucial to get the unique flavor profile and refreshing character of most sour beers (Van Oevelen et al., 1976; De Roos et al., 2019; Bongaerts et al., 2021).

### Maltooligosaccharide Degradation

Maltooligosaccharides (MOS) is the overarching term of linearly 𝛼-1-4 glycosidically bound glucopyranosyl units, covering chain lengths of two up to 10 glucose molecules. MOS are formed from starch due to a breakdown by heat and endogenous amylase activity, mainly during the mashing process (Fangel et al., 2018). During beer production with pitching of an axenic yeast culture of *S. cerevisiae*, *S. pastorianus* or *S. bayanus*, MOS remain untouched, due to the lack of the expression of the degrading enzymes (Sheih et al., 1979). Other yeast species, such as *Saccharomyces kudriavzevii* but especially *Brettanomyces/Dekkera* spp., are known to express 𝛼-glucosidases, which allows the metabolism of MOS (Kumara et al., 1993; De Roos et al., 2020). Especially *Brettanomyces/Dekkera* spp. can metabolize this additional substrate when all mono- and disaccharides are depleted and hence explaining their growth and activity during the maturation phase of lambic beer production (De Roos and De Vuyst, 2019; Bongaerts et al., 2021). Besides MOS breakdown by yeasts, shotgun metagenomic research of fermenting lambic beer wort has shown that two genes for MOS breakdown, encoding maltooligosyl trehalose synthase and maltooligosyl trehalose, are associated mainly with *A. pasteurianus* as well as with *Acetobacter pomorum* and an unknown *Acetobacter* species, most likely *A. lambici* (De Roos et al., 2020). Shotgun metagenomic research of fermenting lambic beer wort has additionally demonstrated that *A. cerevisiae* and *Acetobacter malorum* contain these two genes, encoding maltooligosyl trehalose synthase and maltooligosyl trehalose, as well (De Roos et al., unpublished results). These two enzymes allow the degradation of MOS by their conversion into maltooligosyl trehalose, followed by the release of trehalose (disaccharide of 𝛼-1-1 glycosidically bound glucopyranosyl molecules) by maltooligosyl trehalose activity, to protect the cells against osmotic stress (Zhang et al., 2015; De Roos et al., 2020). Although these two genes are present, it still has to be confirmed if MOS degradation indeed takes place by the latter two species, since high acetic acid concentrations inhibit the biosynthesis of trehalose (Yang et al., 2019). Surprisingly, examining the whole genome of one of the most encountered AAB species, *A. lambici*, it is not one of the species possessing the latter two enzymes for MOS degradation *via* trehalose, although it is very well adapted to the harsh late stages of the lambic beer production process when all mono- and disaccharides are depleted (De Roos et al., 2020; De Roos et al., unpublished results).

### Ester Formation

Esters are extremely important for flavor formation of fermented beverages, including beer, among which ethyl acetate, isoamyl acetate, ethyl hexanoate and ethyl octanoate are the most desired ones produced by yeasts (Engan, 1974; Verstrepen et al., 2003; He et al., 2014). The ester profile of sour beers covers two main types, namely ethyl esters (the condensation products of ethanol and fatty acids) and acetate esters (the condensation products of acetic acid and higher alcohols; Pires et al., 2014; Bongaerts et al., 2021). The ester profile of sour beers differs from top-fermented and bottom-fermented beers, as AAB species contribute to not only acetic acid formation but also ester formation by their expression of esterases (Kashima et al., 2000; De Roos et al., 2020). AAB, more specifically *Acetobacter* spp., possess the intracellular esterases EST1 and EST2 and are thus able to catalyze the condensation of ethanol and acetic acid into ethyl acetate, the most abundant ester in lambic beers (Van Oevelen et al., 1976; Kashima et al., 1998, 2000; Tonsmeire, 2014; Witrick et al., 2017; De Roos and De Vuyst, 2019; De Roos et al., 2020; Bongaerts et al., 2021). Ethyl acetate is of indisputable importance for the lambic beer flavor, and by extension sour beer flavor, due to its high odor activity value and high concentrations. Its formation is ascribed to not only the activities of *Brettanomyces* yeasts and AAB but also chemical esterification of ethanol and acetate (Van Oevelen et al., 1976; Verstrepen et al., 2003; De Roos et al., 2018a). Besides with the formation of ethyl acetate, esterase EST1 is also linked with the formation of isoamyl acetate, an abundantly formed ester by *S. cerevisiae* (Kashima et al., 2000).

### Acetoin Production

Acetoin or 3-hydroxy-2-butanone is a flavor-active compound associated with yoghurt flavor, cream odor, and buttery taste (Xiao and Lu, 2014; De Roos and De Vuyst, 2018, 2019; Bongaerts et al., 2021). Acetoin can be produced by yeasts, LAB (such as *P. damnosus*) and AAB, since they all possess the necessary genes. *Acetobacter* spp. are most likely the main producers of acetoin in sour beers, as has been shown during lambic beer productions and cocoa fermentation processes (Adler et al., 2014; Moens et al., 2014; De Roos et al., 2018a,b, 2020). The acetoin concentrations increase when AAB appear and acetoin is produced more at the air/liquid interface of fermenting lambic beer wort in wooden barrels, where typically AAB are present in higher numbers (De Roos et al., 2018a,b, 2020). Indeed, lactic acid can be used as carbon source and oxidized into pyruvate, further converted by acetolactate synthase and/or acetolactate decarboxylase into 𝛼-acetolactate and finally by diacetyl reductase into acetoin (Adler et al., 2014; Moens et al., 2014; De Roos et al., 2020; Pelicaen et al., 2020). These features have been supported by metagenomic identification of the appropriate genes and phenotypically in both lambic beer productions and cocoa fermentation processes (Moens et al., 2014; De Roos et al., 2020). Acetoin, in combination with acetic acid, contributes to sour beer flavors. However, excessive concentrations should be avoided, since high acetoin levels can cause undesirable buttery notes (De Roos et al., 2018a; Bongaerts et al., 2021). AAB growth should always be controlled by using well-sealed wooden barrels, which do allow microaerobic conditions through the pores of the wood and the formation of a yeast pellicle at the surface of the liquor to limit oxygen inlet from the headspace into the beer. Volume adjustments over time compensate volume losses by evaporation, decrease headspace volumes containing oxygen, and decrease the contact surface between the maturing beer and the barrel headspace, all limiting oxygen entering the beer, and so preventing AAB to grow too extensively (Tonsmeire, 2014; De Roos et al., 2018b, 2019). Finally, the temperature should always be controlled and kept stable, typically below 20°C, again to prevent excessive AAB growth (Van den Steen, 2012; Tonsmeire, 2014).

## Health-Promoting Properties

Sour beers, especially the ones produced by a spontaneous fermentation process, can be linked with health benefits (De Roos and De Vuyst, 2022). First, sour beers containing living LAB and/or AAB cells may contribute to a good microbial balance inside the human gut (Marco et al., 2017, 2021; De Roos and De Vuyst, 2022). Regarding this feature, sour beers have been produced using probiotic LAB strains, for instance with *Lacticaseibacillus paracasei* L26 and DTA-81, and sour beers have been evaluated regarding their suitability as probiotic delivery matrix, which has been demonstrated successfully if produced using a semi-separated co-culture system (Chan et al., 2019; Silva et al., 2020). Further, the occurrence of high-molecular-mass pentosans and ß-glucans, produced by LAB, in sour beer can provide this beer with a natural source of prebiotics (Peyer et al., 2017; Silva et al., 2020).

Regarding caloric values, sour beers can be a helping hand. Whereas non-sour beers are rich in calories, mainly caused by the ethanol content in combination with the presence of residual unfermentable carbohydrates, most sour beers are almost carbohydrate-free, thanks to their complete MOS degradation by interference of non-conventional yeasts, LAB, and/or AAB (De Cort et al., 1994; Briggs et al., 2004; Tonsmeire, 2014; Bossaert et al., 2019; De Roos et al., 2020; De Roos et al., unpublished results). Indeed, if sour beers are produced using *Brettanomyces* yeast species, intentionally added or not, the fraction of unfermentable carbohydrates decreases because of extracellular and intracellular 𝛼-glucosidase activities, causing the breakdown of MOS with chain lengths of at least up to eight glucose molecules (Kumara et al., 1993; De Roos and De Vuyst, 2019; De Roos et al., 2020; Bongaerts et al., 2021).

Finally, regarding antioxidant properties, especially those provided by polyphenolic compounds, sour beers produced by the addition of fresh fruits contain significantly high concentrations of these compounds (Cho et al., 2018; Kawa-Rygielska et al., 2019; Zapata et al., 2019; Nardini and Garaguso, 2020). Fruit addition acts as additional source of antioxidant compounds (e.g., carotenoids, tocopherols, and/or ascorbic acid), besides those provided by barley and hops (Nardini and Garaguso, 2020). Beer antioxidant activities and polyphenolic contents are influenced by the raw materials used, the quantity and quality of the fruits added, and the brewing process applied (Kawa-Rygielska et al., 2019; Nardini and Garaguso, 2020). Dietary intake of antioxidants counters negative effects of oxidative stress, which is caused by the overproduction of reactive oxygen species or reactive nitrogen species (Martinez-Gomes et al., 2020).

## Conclusion

Historically, the role of AAB in beers was underestimated and limited to solely oxidation of ethanol into acetic acid, causing a sharp sour taste and pungent smell, impacting the flavor of most beers negatively. Despite their negative imago, research has extended the knowledge about AAB, exposing new features of AAB in sour beers. Sour beer production involving AAB can possibly result in more complex and funky beers. To achieve a positive contribution of AAB to the beer flavor, controlled growth should always be aimed for. Despite an increased understanding of AAB and their functional role during sour beer production, controlled growth of AAB with sufficient but not excessive production of flavor compounds, such as acetic acid and acetoin, requires skills only experienced brewers do master.

## Author Contributions

AB drafted the manuscript. AB and LDV revised and edited the manuscript. All authors contributed to the article and approved the submitted version.

## Funding

This work was supported by the Research Council of the Vrije Universiteit Brussel (SRP7 and IOF3017 projects).

## Conflict of Interest

The authors declare that the research was conducted in the absence of any commercial or financial relationships that could be construed as a potential conflict of interest.

## Publisher’s Note

All claims expressed in this article are solely those of the authors and do not necessarily represent those of their affiliated organizations, or those of the publisher, the editors and the reviewers. Any product that may be evaluated in this article, or claim that may be made by its manufacturer, is not guaranteed or endorsed by the publisher.
